# Mitochondrial Translocation of Vitamin D Receptor Is Mediated by the Permeability Transition Pore in Human Keratinocyte Cell Line

**DOI:** 10.1371/journal.pone.0054716

**Published:** 2013-01-22

**Authors:** Francesca Silvagno, Marco Consiglio, Valentina Foglizzo, Michele Destefanis, Gianpiero Pescarmona

**Affiliations:** 1 Department of Oncology, University of Torino, Torino, Italy; 2 Center for Experimental Research and Medical Studies, S. Giovanni Battista Hospital, Torino, Italy; Nihon University School of Medicine, Japan

## Abstract

**Background:**

Vitamin D receptor (VDR) is a well known transcriptional regulator, active as heterodimer in association with coactivators and corepressors. In addition it has been described the extranuclear distribution of the receptor and in particular the recently reported mitochondrial localization in platelets and megakaryocytes is intriguing because it appears to be a common feature of steroid receptors. Whereas for other members of the steroid receptor family the mitochondrial function has been explored, up to now nothing is known about a mitochondrial form of VDR in human proliferating cells.

**Methodology/Principal Findings:**

In this study we characterized for the first time the mitochondrial localization of VDR in the human keratinocyte cell line HaCaT. In proliferating HaCaT cells VDR was abundantly expressed in mitochondria in association with its binding partner RXRα and the import was ligand-independent. By immunoprecipitation studies we demonstrated the interaction of VDR with proteins of the permeability transition pore (PTP), VDAC and StAR. We then adopted different pharmacological and silencing approaches with the aim of hampering PTP function, either affecting PTP opening or abating the expression of the complex member StAR. By all means the impairment of pore function led to a reduction of mitochondrial levels of VDR.

**Conclusions:**

The results reported here demonstrate a ligand-independent mitochondrial import of VDR through the permeability transition pore, and open interesting new perspectives on PTP function as transporter and on VDR role in mitochondria.

## Introduction

Vitamin D (the active form 1,25-dihydroxyvitamin D3, 1,25D3) exerts its antiproliferative and differentiating properties through its receptor (VDR). Like other members of the steroid hormone receptors family, VDR is well known as transcriptional factor and carries on its function upon nuclear translocation, heterodimerization with RXRα and association with coactivators or corepressors to VDRE regions of DNA. VDR activity is controlled by p53, which both induces receptor expression and synergizes with it at VDRE sites [1]. With this mechanism, for example, p53 and VDR trigger cell cycle arrest through p21 (waf1/cip1) action [2]. Besides their classical nuclear function for many steroid receptors a non genomic signalling pathway has been described, either plasma membrane bound or at mitochondrial level. Steroid receptors such as RXRα, thyroid receptor (TR), glucocorticoid receptor (GR), estrogen receptor (ER) show an additional mitochondrial localization which suggests a role either in mitochondrial biogenesis or metabolism [3]–[5]. Many nuclear transcription factors may participate in regulating mitochondrial function through transcriptional regulation of mitochondrial DNA [6].

Also p53 can translocate to mitochondria, where it is involved in apoptosis. The transcription independent pathway of p53 mediated apoptosis has been described as a destabilization of the outer mitochondrial membrane by complexing with the anti-apoptotic proteins Bcl-xL and Bcl2 and by activation of the cytoplasmic proapoptotic protein Bax [7].

Apart from our previous work on human platelets and megakaryocytes, where we described a VDR located in mitochondria [8], there are very few evidences of a mitochondrial localization for VDR, presented as mere observations [9]–[11]. The aim of this work was therefore to characterize VDR subcellular distribution in proliferating human cells. We carried out our study on a cellular model highly expressing VDR, the HaCaT cell line, a spontaneously transformed human epithelial cell line from adult skin which is immortal but maintains full epidermal differentiation capacity [12]. Human keratinocytes not only respond to vitamin D with changes in proliferation and differentiation but also synthesize vitamin D and its metabolites [13]. HaCaT cells express nuclear VDR and have been used as a model for the investigation of the vitamin D pathway and its modulation [14].

In this study, we discovered that VDR, besides its nuclear expression, can localize also in the mitochondria of human proliferating cells and that VDR mitochondrial import requires permeability transition pore (PTP) stability and functioning. These observations provide new insight into VDR localization and put forward a novel mechanism whereby a receptor can be imported into the mitochondria.

## Materials and Methods

### Cell Culture and Treatments

Immortalized human epidermal keratinocytes (HaCaT) were purchased from American Type Culture Collection (ATCC), USA, and were cultured in Dulbecco’s modified Eagle’s medium (DMEM) supplemented with 10% fetal bovine serum and 1% antibiotics [penicillin-streptomycin (Sigma-Aldrich)] at 37°C in humidified 5% CO_2_ atmosphere. When treated, cells were kept in the same medium supplemented with 1% fetal bovine serum. Cells were incubated for reported time with 1 or 100 nM 1,25(OH)_2_ vitamin D3 (Sigma), 10 µM squalestatin (zaragozig acid, Sigma), 5 µg/ml cycloheximide, 5 µM cyclosporine A, 100 nM dexamethasone, with periodical replacement of medium. Latter drugs were from Calbiochem, La Jolla, CA. Other reagents were from Sigma.

### Primary Antibodies

As previously described [8], the following antibodies against VDR were used: rabbit polyclonal anti-VDR (C-terminus fragment) clone C-20 (sc-1008, Santa Cruz Biotechnology, CA); rat monoclonal anti-VDR biotin labeled (aa 89–105 epitope) clone 9A7c.E10.E4 (RT-200-B, LabVision NeoMarkers, CA). The antibody against RXRα was commercially available from Santa Cruz (rabbit antibody D-20 against N-terminus of human RXRα), whereas the biotinylated form was made in our laboratory conjugating biotin to mouse antibody against aa 198–462 of human RXRα (clone F-1, Santa Cruz). Briefly, after removing gelatin from IgG, the antibody was labeled with biotin by EZ-Link Micro Sulfo-NHS-Biotinylation kit (Pierce) following the indications of the manufacturer. Sulfo-NHS-LC-Biotin was used in labeling reaction, followed by excess biotin removal and storage of biotinylated antibody in 2 mM BSA, 0,02% Na Azide.

Monoclonal antibody anti-VDAC (anti-porin 31HL) was purchased from Calbiochem. Mouse monoclonal antibodies anti-actin (sc-8432) and anti-StAR (sc-166821) and rabbit antibody anti-PARP (sc-7150) were from Santa Cruz, CA, USA.

### Extracts Preparation

Subcellular fractionation was carried out following the procedure described by Capiati et al. [15], with some modifications. Cells were scrapped from the dishes and homogenized in a glass hand homogenizer in 10 mM Tris-HCl, pH 7.4, 0.33 M sucrose, 1 mM EDTA, 1 mM dithiothreitol, 1 mM phenylmethylsulphonyl fluoride (PMSF), 1 mM protease inhibitors Cocktail set III (Calbiochem), 1 mM Na_3_VO_3_, 1 mM NaF. An aliquot was kept for protein analysis (total extract) before the lysates were subjected to differential centrifugation, first at 200 g for 10 min to eliminate cell debris, followed by 1,000 g for 10 min to isolate the nuclear fraction, and 14,300 g for 20 min to obtain a soluble fraction as supernatant and the mitochondrial fraction as a pellet. The latter was resuspended in boiling sample buffer (SB 1X: 100 mM Tris–HCl, pH 6.8, 0.01% bromophenol Blue, 2% SDS, 15% glycerol, 10 mM DTT). Proteins were extracted from nuclei by incubation in boiling sample buffer followed by sonication. Nuclear debris was finally centrifuged for 15 min at 16,000×g at 4°C and supernatants, corresponding to nuclear extracts, were kept for protein analysis. All fractions were quantified for protein content by DC protein assay Biorad.

### Immunoprecipitation and Western Blotting

For immunoprecipitation studies, the mitochondrial fraction obtained as described above was resuspended in RIPA buffer (10 mM Tris pH 7.4, 150 mM NaCl, 1% NP-40, 0.5% sodium deoxycholate, 0.1% SDS, 1 mM protease inhibitors, 1 mM PMSF). 300 µg of proteins were incubated with rotation overnight at 4°C with 1 µg of antibody, followed by an additional incubation for 2 h with protein G agarose beads. Proteins were collected by centrifugation after boiling in SB 1x. All subcellular fractions and immune complexes were separated by 10% SDS-PAGE and then transferred overnight to PVDF membranes (Immobilon-P, Millipore, Bedford, MA). Western blotting analysis was performed as described previously [16]. Proteins were immunostained with the indicated primary antibodies for 1 h at room temperature. Detection of protein of interest was performed using peroxidase-conjugated secondary antibodies (Pierce, Rockford, IL) followed by ECL detection (ECL detection kit, Perkin Elmer Life Science, Boston, MA, USA). Because of the similar molecular weight of VDR, RXRα and IgG heavy chain, the identification of VDR and RXRα from immunoprecipitates was carried out with the biotinylated 9A7c primary antibody and biotinylated RXRα antibody respectively, followed by a streptavidin-HRP incubation and ECL detection.

### Lentivirus Production and Cellular Transduction

PLKO.1 lentiviral vectors expressing the shRNAs against human VDR (TRCN0000019506), human StAR (TRCN0000040223) and the non-target control shRNA (SHC002) were purchased from SIGMA Aldrich. Lentiviral transduction particles were produced in HEK293T cells by co-transfection of either the control or the human VDR or StAR shRNA plasmid together with packaging vectors pMDLg/pRRE, pRSVRev, and pMD2.VSVG. Lipofectamine 2000 (LifeTechnologies) was used as transfection reagent. Supernatants were harvested 30 hours after transfection, filtered through 0.22-Am pore size filters (Corning Science Products) and used directly for over/night trasduction of HaCaT keratinocytes. Puromycin selection started 24 hours after HaCaT infection. Five days after infection cells were harvested, mitochondria and nuclei were separated as described above and proteins from different fractions were analysed by western blotting for StAR and VDR expression levels.

### Bands Quantification and Statistical Analysis

Bands from protein electrophoresis were quantified by scanning digital densitometry using an ImageJ software analysis (ImageJ version 1.29, Sun Microsystems Inc., Palo Alto, CA). Statistical analysis of data was performed using ANOVA test with Tukey’s post-hoc correction. p values <0.05 were considered significant and indicated when treatments were compared to control. All data were expressed as mean ± S.D.

## Results

### HaCaT Cells Express a Mitochondrial form of VDR and RXRα

**Figure 1:**
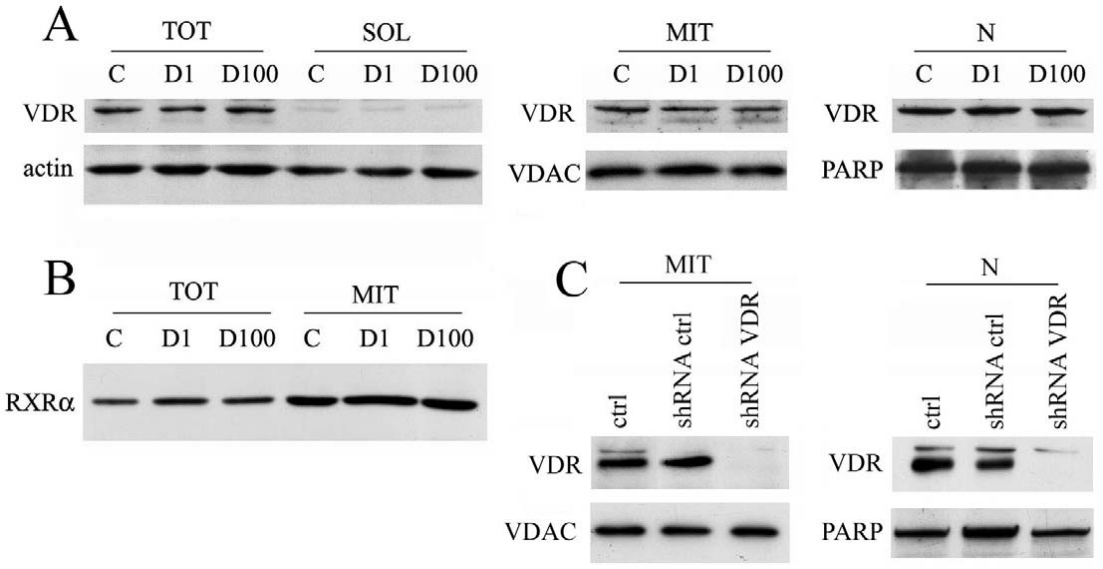
VDR expression and subcellular distribution in HaCaT cells. Cell were incubated for 24 hours alone (control, C) or with 1 or 100 nM 1,25D3 (D1 or D100) and harvested. (A) After subcellular fractionation procedures 50 µg of proteins from total extracts (TOT) and soluble fraction (SOL), and 10 µg of proteins from mitochondria (MIT) and nuclear extracts (N) were separated by SDS-PAGE and analysed by western blotting for VDR expression. Equal loading and quality of samples was confirmed by reprobing the membranes with antibodies anti actin, VDAC (mitochondrial marker) and PARP (nuclear marker). (B) Same amount of total extracts and mitochondrial fractions were analysed by western blotting for RXRα expression. (C) 10 µg of proteins from mitochondria (MIT) and nuclear extracts (N) of untreated cells (ctrl) or cells infected with shRNA control and shRNA anti-VDR were analysed by western blotting for VDR expression and afterwards for loading uniformity.

First of all we decided to characterize the subcellular distribution of the receptor in HaCaT cells untreated or treated with 1 nM or 100 nM 1,25-dihydroxyvitamin D3 for 24 hours. Total extracts were fractionated by differential centrifugation in soluble, mitochondrial and nuclear preparations and each fraction was analysed by western blotting. The rabbit polyclonal antibody C-20 recognized with specificity VDR protein at the reported molecular weight of about 50 kDa, as demonstrated in our previous work [8]. As shown in figure 1A, VDR was evident in total lysates and the fractionation procedure led to the intensification of receptor expression both in nuclear extracts and in the mitochondrial fraction. Mitochondrial and nuclear enrichment was evaluated by analysis of VDAC in mitochondrial fraction and PARP in nuclear samples. VDR expression was not affected by vitamin D treatment at two different concentrations. The analysis of the same samples revealed also a strong mitochondrial expression of RXRα (fig. 1B). Mitochondrial and nuclear VDR expression was confirmed by knockdown experiments. Upon silencing of the receptor by lentiviral shRNA delivery the protein disappeared from mitochondrial and nuclear extracts, as shown in figure 1C.

### VDR Associates to the Permeability Transition Pore (PTP) Components

**Figure 2:**
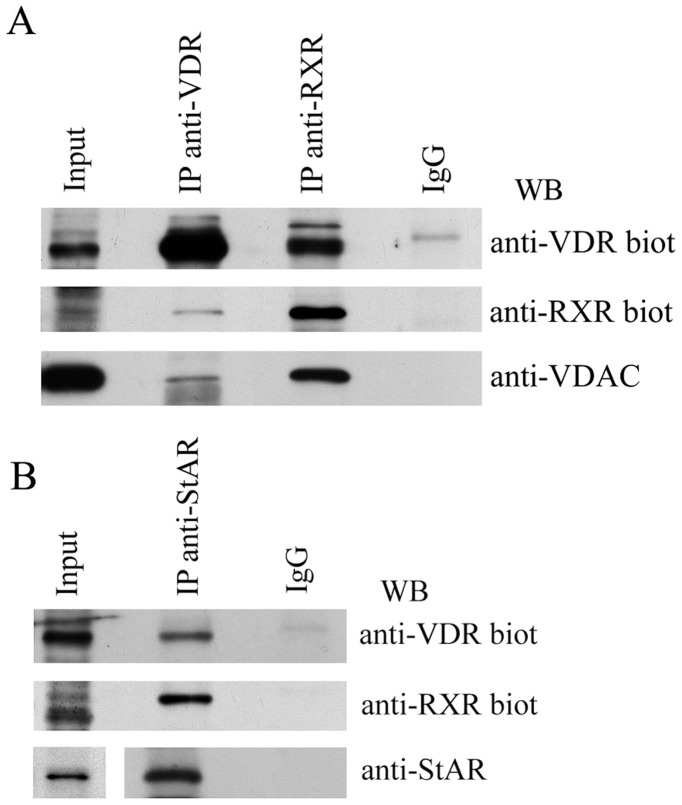
VDR association with RXRα and PTP proteins. (A) Mitochondrial extracts from untreated HaCaT cells were immunoprecipitated with anti-VDR and anti-RXRα rabbit antibody and detection by western blotting was performed with anti-VDR or anti-RXRα biotinylated antibodies and with anti-VDAC rabbit antibody. (B) The interaction between VDR, RXRα and StAR was investigated in mitochondrial fraction by immunoprecipitation with anti-StAR rabbit antibody followed by western blotting and detection with anti-VDR and anti-RXRα biotinylated antibodies. In every assay a 10% input was used as a positive control and immunoprecipitation with normal IgG as negative control.

In order to investigate the mechanism of mitochondrial import of VDR, we hypothesized that VDR translocation could be mediated by the classical import machinery known as TOM/TIM translocase. However, upon analysis by protein subcellular localization prediction methods (Mitoprot II, TargetP 1.1, Predotar, iPSORT, WoLFPSORT) we could not find any import sequence at the N-terminal of the receptor. Therefore we took into consideration the possibility that VDR might cross the mitochondrial membranes through the permeability transition pore, a complex up to now little investigated as import channel. To verify this possibility we performed some experiments aimed at demonstrating the interaction between VDR and the proteins of PTP. We chose to investigate a possible association of VDR with VDAC and StAR, two components of the outer membrane layer of PTP. By immunoprecipitation studies we found that in the mitochondrial fractions VDR interacted with RXRα, as reasonably expected since RXRα is a known binding partner of VDR (fig. 2A). Most interesting, we obtained the evidence that VDR and RXRα associated with VDAC, as shown in figure 2A, and StAR, as demonstrated by figure 2B.

### Blocking the Opening of PTP Hampers VDR Mitochondrial Import

**Figure 3:**
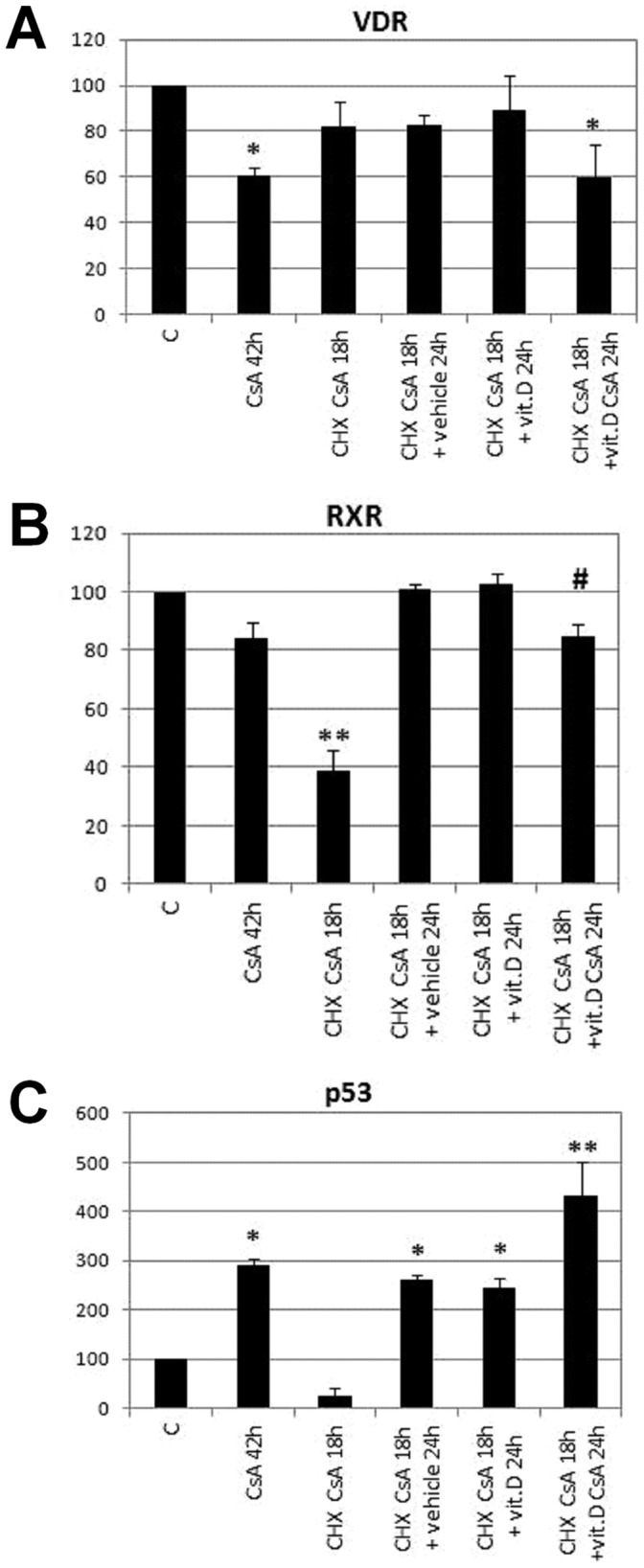
Analysis of mitochondrial translocation of VDR in presence of cyclosporin A. HaCaT cells were treated with cyclosporin A (CsA), cycloheximide (CHX) or 100 nM 1,25D3 (Vit.D) as indicated and 30 µg of mitochondrial proteins were analysed by western blotting for VDR, RXRα and p53 expression. Bands were quantified, normalized for loading as a ratio to VDAC expression and data plotted on graph as percentage of control. Data represent the mean ± S.D of three independent experiments. *p<0.05 and **p<0.001 compared to control. # *p*<0.05 vs CHX CsA 18 h+vit.D 24 h.

We set up experimental conditions able to demonstrate that closing PTP interfered with VDR mitochondrial translocation. We treated HaCaT cells for 18 hours with an inhibitor of protein synthesis (cycloheximide, CHX) and an inhibitor of PTP function (cyclosporin A, CsA) [17]. The combined treatment allowed us to distinguish two mitochondrial protein pools, because CsA sequesters into mitochondria proteins dependent solely on PTP for their translocation. In presence of CsA, CHX-insensitive pool is made of proteins confined into mitochondria and therefore insensitive to synthesis inhibition, whereas CHX-sensitive proteins are synthesized and imported by TOM/TIM translocase [18]. After the cotreatment CHX was washed out and cells were incubated for additional 24 hours with or without CsA in presence or absence of vitamin D. We analysed protein content of mitochondrial fractions by western blotting and evaluated the expression of VDR, RXRα and p53, which are proteins able to translocate to mitochondrial compartment by different mechanisms. All values were normalized for VDAC expression, which was constant in all conditions, and results are shown in figure 3. Representative images of blots are displayed in figure S1. As demonstrated by graph in fig. 3A, 42 hours of treatment with CsA resulted in a marked reduction of VDR, and allowed an evaluation of a t_1/2_ of 48 hours for mitochondrial VDR. Cotreatment with CHX and CsA for 18 hours decreased only slightly and not significantly mitochondrial import of VDR, and only if the cotreatment was followed by incubation with CsA for further 24 hours VDR import was hampered, whereas if vit. D or medium alone was added VDR import was similar to control. These experiments demonstrated that VDR import is CHX insensitive and CsA dependent, and therefore mediated by PTP. In the same conditions quantification of mitochondrial RXRα gave different results, as shown by graph in fig. 3B. Mitochondrial import of this receptor was CHX-dependent; in fact it was only slightly altered in presence of CsA alone whereas it strongly decreased upon cotreatment of CsA with CHX for 18 hours. Eliminating CHX from incubation medium in the following 24 hours restored mitochondrial content of RXRα but in presence of CsA to a significantly lesser extent. We therefore reached the conclusion that mitochondrial import of RXRα CHX-dependent and mildly sensitive to CsA, was mainly mediated by a translocase. Finally, analysis of mitochondrial p53 presented in graph 3C revealed a strong decrease in presence of CHX, which is in agreement with the short half-life of the protein, whereas CsA had the opposite effect on the protein, increasing mitochondrial p53.

### Squalestatin Decreases StAR Expression and VDR Mitochondrial Import

**Figure 4:**
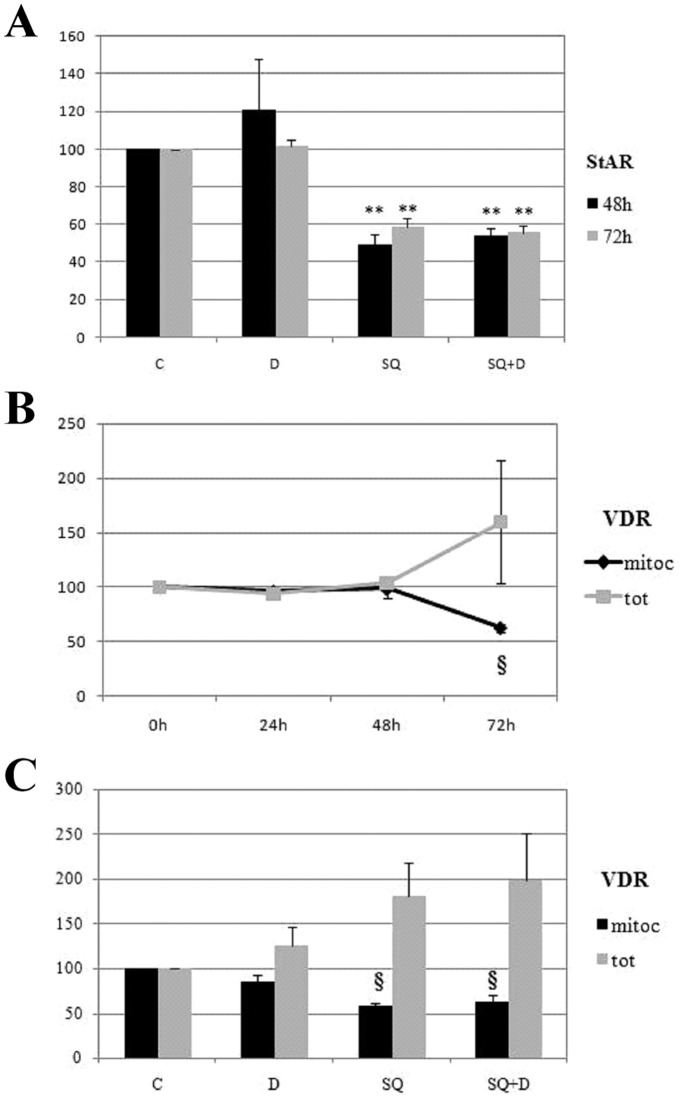
Effect of squalestatin treatment on StAR and VDR expression. HaCaT cells were treated with squalestatin (SQ) and 100 nM 1,25D3 (D) or left untreated (control, C), harvested and 50 µg of total lysates (tot) or 30 µg of mitochondrial proteins (mitoc) were analysed by western blotting for StAR and VDR expression. Bands were quantified, normalized for loading as a ratio to actin expression (tot) or VDAC expression (mitoc) and data plotted on graph as percentage of control. Data represent the mean ± S.D of three independent experiments. (A) Analysis of StAR expression in mitochondrial fractions after 48 or 72 hours of treatment with SQ, 24 hours with vit. D or cotreatment in which vit. D was added in the last 24 hours (SQ+D). (B) VDR expression after squalestatin treatment in a time course experiment. (C) VDR expression after 72 hours of the same treatments as in (A). ^§^ *p*<0.01 and **p<0.001 compared to control.

The second pharmacological approach aimed at affecting PTP functioning was down-regulating the expression of StAR, one key protein of the complex. StAR was first characterized as mitochondrial transporter of cholesterol in cells active in steroids biosynthesis, and in second instance it was recognized as a member of the PTP protein cluster. We reasoned that StAR expression could be induced by substrate availability and decreased by substrate shortage, in similarity with other proteins involved in handling intracellular cholesterol such as ACAT (acyl-coenzyme A:cholesterol acyltransferase) [19] and the cholesterol transporter ABCA1 [20]. We used squalestatin to inhibit endogenous cholesterol biosynthesis and we analysed StAR expression by western blotting. As shown in figure 4A the analysis of mitochondrial StAR revealed a decrease of about 50% after a treatment with squalestatin for 48 and 72 hours, whereas treatment with vit. D did not affect StAR expression. We then carried out the quantification of VDR expression in presence of squalestatin in a time course experiment as shown in figure 4B and we found that mitochondrial VDR decreased only after 72 hours of incubation with the drug. In the same conditions VDR expression analysed in total extracts was never affected, leading to the conclusion that the decrease of StAR had no direct effect on VDR expression, but instead it hampered VDR mitochondrial import by PTP. Finally we checked whether in the last 24 hours of squalestatin treatment the presence of vit. D could make a difference in receptor import, but it did not, since mitochondrial VDR decreased by squalestatin did not change if vit. D was added, as demonstrated by graph in figure 4C. Representative images of blots are displayed in figure S2.

### Dexamethasone Elicits a Decrease of Mitochondrial StAR and VDR

**Figure 5:**
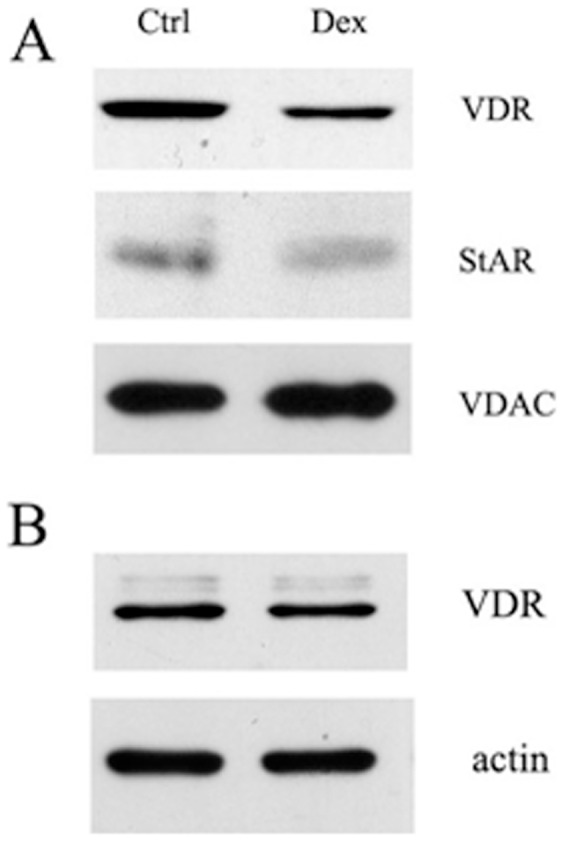
Western blot analysis of the expression of VDR and StAR upon dexamethasone treatment. 30 µg of mitochondrial proteins (A) or whole lysates (B) from untreated HaCaT cells (ctrl) and cells treated for 72 h with dexamethasone (Dex) were analysed by western blotting using an antibody anti-VDR, followed by immunostaining with anti-StAR and finally with anti-VDAC or anti-actin antibody for loading control. The blots are representative of a set of three independent experiments.

We realized that an alternative treatment able to decrease mitochondrial StAR expression was the incubation with dexamethasone (dex), as previously published [21]. We therefore used dex to confirm the decreased mitochondrial import of VDR seen in presence of squalestatin. Given the stability of mitochondrial VDR, an incubation of 72 hour with dex was carried out and the experiment led to a decrease of mitochondrial StAR along with mitochondrial VDR, whereas total VDR expression was not affected, as shown in figure 5. We concluded that dexamethasone brought about a StAR-dependent reduced mitochondrial import of VDR.

### Genetic Silencing of StAR Reduces VDR Mitochondrial Import

**Figure 6:**
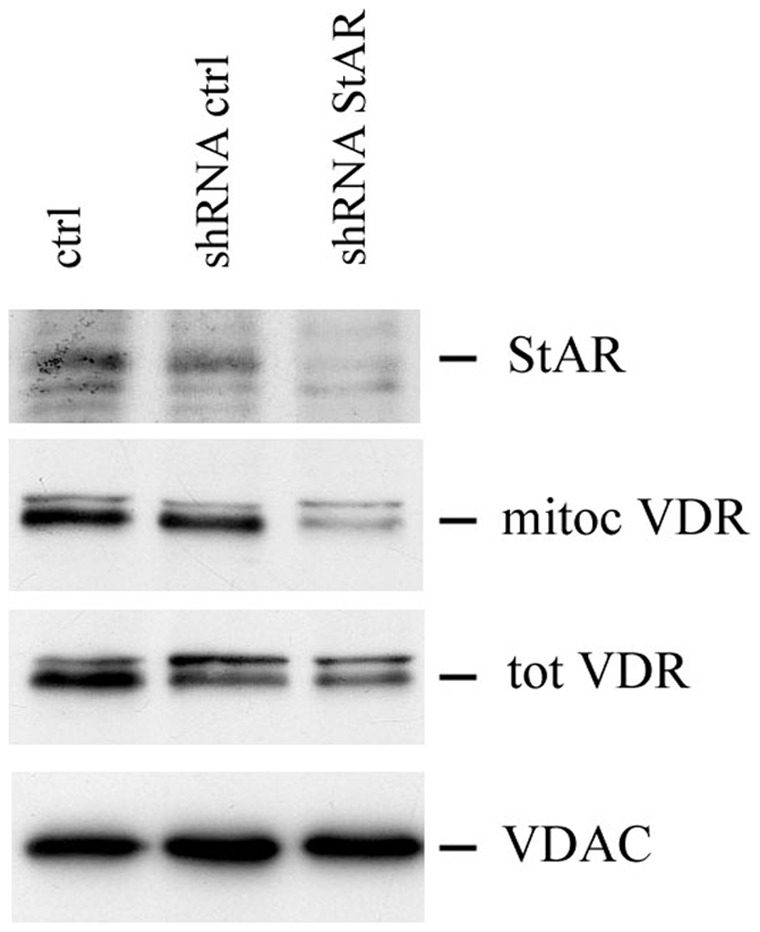
Effect of genetic silencing of StAR on VDR expression. Subconfluent HaCaT cells were infected with lentiviral StAR shRNA particles to silence the endogenous StAR expression. Mitochondrial fractions from untreated HaCaT (ctrl) and cells infected with shRNA control and StAR were analysed by western blotting for StAR and VDR expression. VDR levels were also evaluated in total lysates. VDAC was used as internal control for protein loading.

In order to confirm the physiological relevance of StAR as mediator of VDR mitochondrial import and to reinforce the data obtained by pharmacological treatments, we used the lentiviral delivery system carrying the StAR-specific shRNA to silence the endogenous expression of StAR in HaCaT cells. We found that consistently with the results displayed in figures 4–5 silencing of endogenous StAR impaired the expression of mitochondrial VDR. As shown in figure 6, five days after infection StAR expression was abated. Its depletion had severe repercussions on mitochondrial localization of VDR, whereas it did not interfere with total VDR protein expression.

## Discussion

Few previous reports suggested the extra-nuclear intracellular distribution of VDR [9]–[11]. Among them our work on platelets and megakaryocytes presented the evidences of a mitochondrial VDR [8]. In this paper we now demonstrate the mitochondrial localization of VDR also in a human proliferating cell line. Human keratinocytes are not only able to synthesize vit. D but also they depend on vit.D/VDR for proliferation and differentiation [22], [23]. We therefore chose to study VDR in the human keratinocytes HaCaT cell line and besides the classical nuclear localization we found an abundant mitochondrial expression of the receptor as well as RXRα its binding partner. VDR distribution was further demonstrated by silencing experiments and was not ligand-dependent: the amount of receptor found in mitochondria was high and not modified by vitamin D. We could define mitochondrial VDR as a resident protein, in contrast with nuclear VDR which is described as a ligand-dependent imported transcription factor. Our work was then focused on identifying the mechanism of VDR mitochondrial import. Since by the most used protein subcellular localization prediction methods we could not find any obvious import sequence at the N-terminal of the receptor, we considered improbable the import through TOM/TIM translocase and we investigated another still underestimated access route to mitochondria: the permeability transition pore (PTP). This channel is formed by a large and dynamic complex of proteins (reviewed in [24]), its opening is voltage-dependent and it is more known for being involved in release of mitochondrial proteins such as cytochrome C during apoptosis, in translocation of nucleotides through the complex member adenine nucleotide translocator ANT and in the transport of cholesterol via another complex member, StAR. However very few reports have shown the interaction of mitochondrial proteins with the components of the channel, suggesting the possibility of the import of proteins larger than 1.5 kDa via the PTP. For example the tumor necrosis factor receptor p75NTR was found in mitochondria where interacts with ANT [25]. Interestingly, there are reports of a link between PTP opening and mitochondrial localization of proteins. For example mitochondrial localization of p-glycoprotein (pgp) is sensitive to selective opener (Atractyloside glycoside, ATR) and inhibitor (Cyclosporin A, CsA) of mitochondrial permeability transition pore [26]. Moreover it has been shown that mitochondrial permeability transition is required for p53 mitochondrial translocation [27].

In order to verify whether PTP could have a role in VDR import, we first demonstrated by immunoprecipitation studies that VDR and RXRα interacted with PTP by association with VDAC and StAR. Then we searched the proofs that such a multimer was functional. We demonstrated that VDR crossed mitochondrial membranes via PTP by three different sets of experiments: a pharmacological inhibition of PTP opening with CsA, a downregulation of StAR triggered by squalestatin and a decreased expression of StAR upon treatment with dexamethasone. Every time we reduced the PTP function mitochondrial levels of VDR were abated. As for the mitochondrial import of RXRα, the immunoprecipitation studies suggested its association with PTP, however the experiments with CsA indicated that the entry via PTP is a somehow minor event and the main mechanism of import is different, because it is CHX sensitive and only slightly modified by CsA. This is in agreement with previous reports [28], [29]. In our experiments mitochondrial p53 was not curtailed by CsA, but on the contrary closing the PTP had an enhancing effect on its mitochondrial localization. These results differ from observations of Liu and coll. [27] because they evaluated p53 import triggered by TPA and after short treatments with CsA. In our hands PTP closure for 42 hours stabilized mitochondrial p53. Taken together the results from CHX-CsA experiments were able to discriminate between proteins crossing mitochondrial membranes via PTP or other mechanisms, and they gave the first indication on how VDR enters mitochondria. Moreover the same experiments were important to demonstrate that mitochondrial VDR is a rather stable receptor, showing a half-time of about 48 hours.

VDR entry via PTP was confirmed by the subsequent experiments that decreased the amount of one member of PTP: StAR. In fact both the treatment with squalestatin and with dexamethasone reduced mitochondrial VDR, reinforcing thus the idea that a well functioning PTP was necessary for mitochondrial compartmentalization of VDR. Since the receptor was abundant and stable, a decline in its mitochondrial presence was visible only after a prolonged inhibition of PTP function (48 hours of closure or 72 hours of lower expression). A confirmation of the data obtained by pharmacological down-regulation of StAR came from silencing experiments. StAR knockdown by lentiviral delivery of shRNA decreased mitochondrial VDR, supporting the role of StAR in the mechanism of VDR mitochondrial import.

The presence of nuclear receptors in mitochondria was already described in literature (reviewed in [6]) and this localization is important for their role in mitochondrial metabolism. Several papers have described the mitochondrial import and function of other steroid receptors, such as RXRα, estrogen receptor α and β, triiodothyronine (T3) receptor, glucocorticoid receptor [30], [31]. For some of them a mitochondrial import signal has been indicated, suggesting a translocase-mediated transport [5] sometimes preceded by partial proteolysis [28], [32]. However in many cases the import mechanism is still obscure. The presented novel mechanism of entry for VDR could apply to some of them.

In conclusion this paper gives for the first time the evidences of VDR mitochondrial localization in human proliferating cells and shows that mitochondrial targeting is mediated by PTP. These findings open interesting future investigations on PTP function as transporter and on VDR role in mitochondria.

## Supporting Information

Figure S1 **Analysis of mitochondrial translocation of VDR in presence of cyclosporin A.** HaCaT cells were treated with cyclosporin A (CsA), cycloheximide (CHX) or 100 nM 1,25D3 (Vit.D) as indicated and 30 µg of mitochondrial proteins were analysed by western blotting for VDR, RXRα and p53 expression. VDAC was used as internal control for protein loading. A representative blot is shown.
(TIF)

Figure S2 **Effect of squalestatin treatment on StAR and VDR expression.** HaCaT cells were treated with squalestatin (SQ) and 100 nM 1,25D3 (D) or left untreated (control, C), harvested and 50 µg of total lysates (tot) or 30 µg of mitochondrial proteins (mit) were analysed by western blotting for StAR and VDR expression. VDAC or actin expression were evaluated as loading control. A set of representative blots is shown. (A) Analysis of StAR expression in mitochondrial fractions. (B) VDR expression after squalestatin treatment in a time course experiment. (C) VDR expression after 72 hours of the same treatments as in (A).
(TIF)
