# Supplementary figures and images for: Mitochondrial Translocation of Vitamin D Receptor Is Mediated by the Permeability Transition Pore in Human Keratinocyte Cell Line
Source: PLoS One. 2013 Jan 22;8(1):e54716. doi: 10.1371/journal.pone.0054716 (PMC3551909; doi:10.1371/journal.pone.0054716)

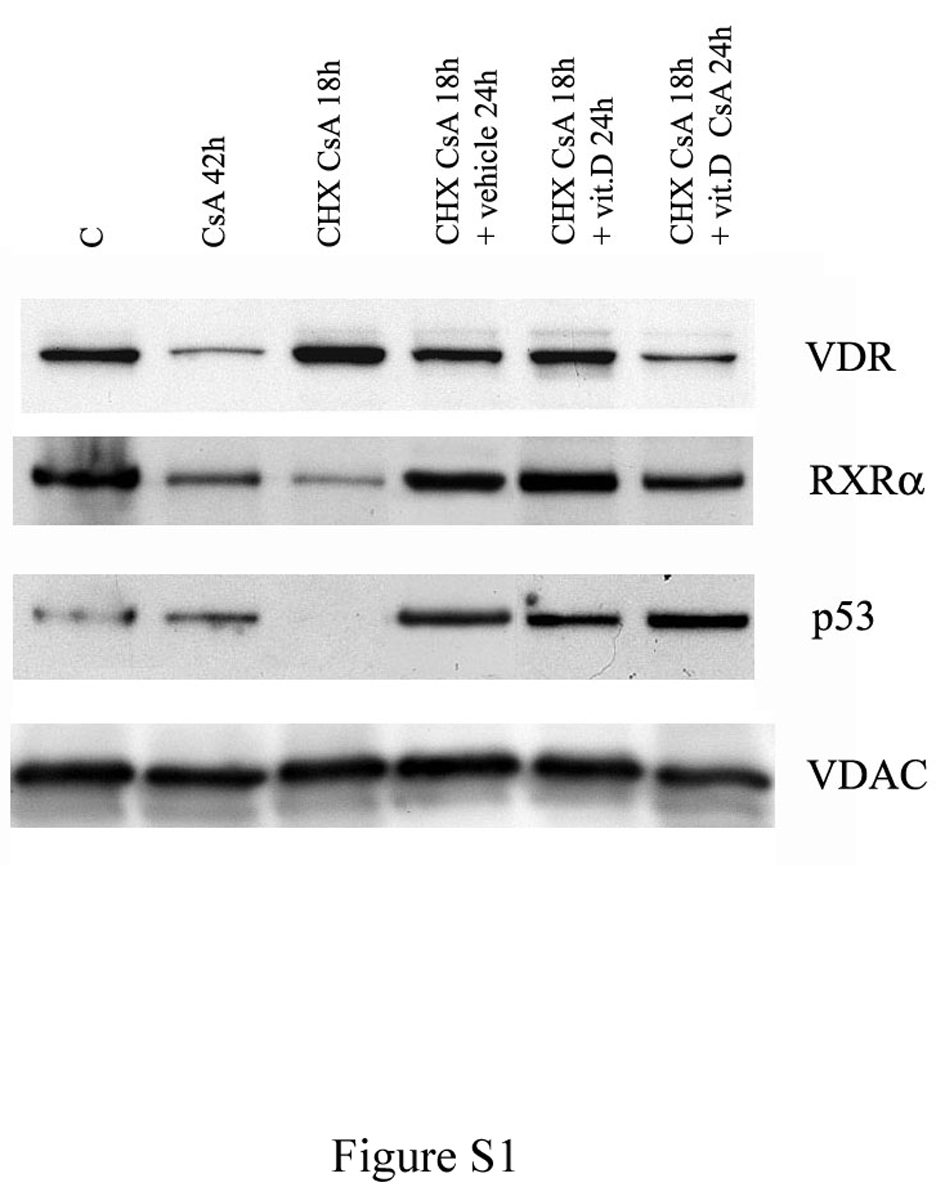

Supplement: Figure S1 — Analysis of mitochondrial translocation of VDR in presence of cyclosporin A. HaCaT cells were treated with cyclosporin A (CsA), cycloheximide (CHX) or 100 nM 1,25D3 (Vit.D) as indicated and 30 µg of mitochondrial proteins were analysed by western blotting for VDR, RXRα and p53 expression. VDAC was used as internal control for protein loading. A representative blot is shown. (TIF) [file pone.0054716.s001.tif]

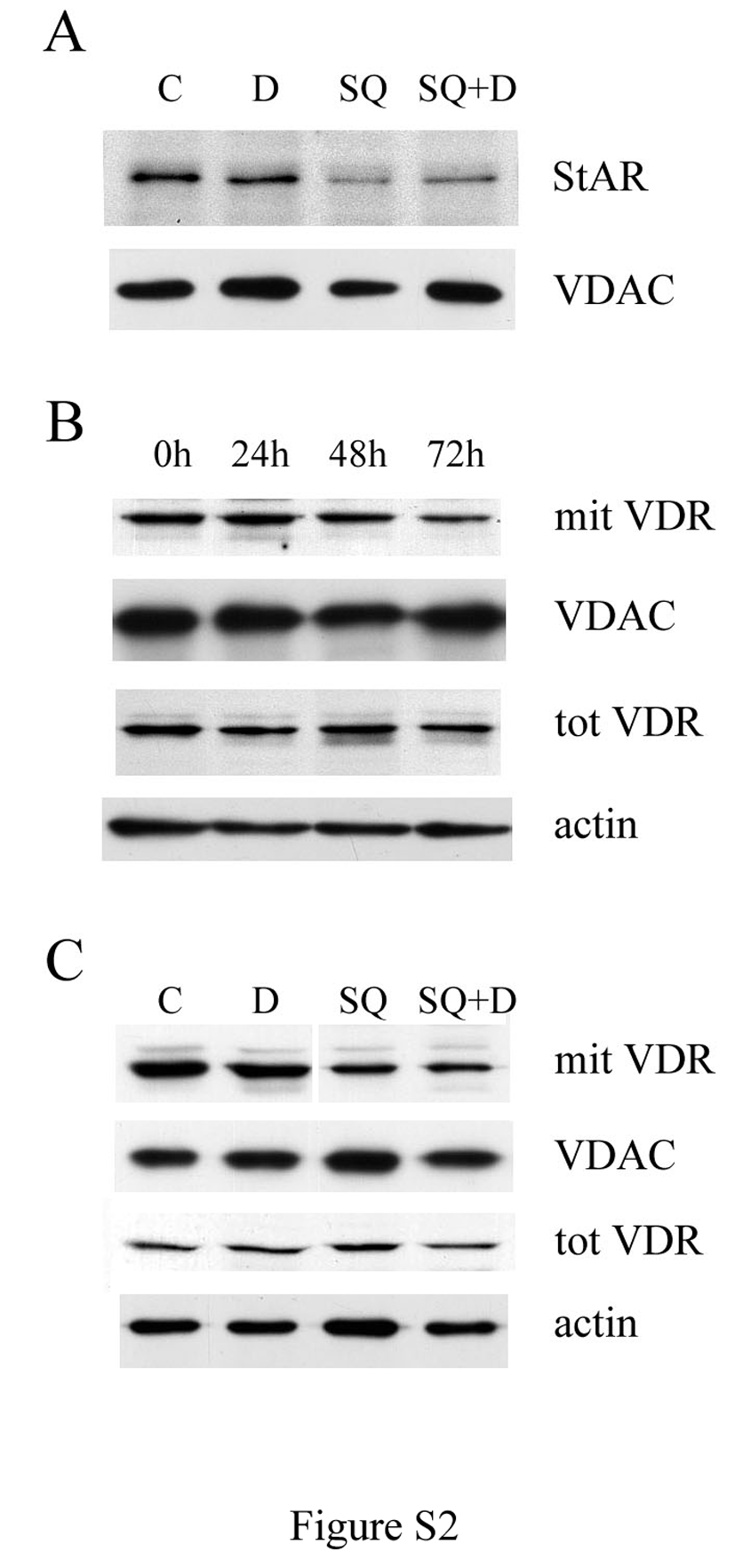

Supplement: Figure S2 — Effect of squalestatin treatment on StAR and VDR expression. HaCaT cells were treated with squalestatin (SQ) and 100 nM 1,25D3 (D) or left untreated (control, C), harvested and 50 µg of total lysates (tot) or 30 µg of mitochondrial proteins (mit) were analysed by western blotting for StAR and VDR expression. VDAC or actin expression were evaluated as loading control. A set of representative blots is shown. (A) Analysis of StAR expression in mitochondrial fractions. (B) VDR expression after squalestatin treatment in a time course experiment. (C) VDR expression after 72 hours of the same treatments as in (A). (TIF) [file pone.0054716.s002.tif]
